# Presence of chromosomal abnormalities in fetuses with isolated ventriculomegaly on prenatal ultrasound in China

**DOI:** 10.1002/mgg3.477

**Published:** 2018-09-19

**Authors:** Dan Zhao, Ailu Cai, Bing Wang, Xiaodan Lu, Lu Meng

**Affiliations:** ^1^ Department of Ultrasound Shengjing Hospital of China Medical University Shenyang China; ^2^ Department of Ultrasound Women's and Children's Hospital Dandong China; ^3^ Department of Ultrasound Women's and Children's Hospital Shenyang China

**Keywords:** chromosomal abnormalities, fetus, prenatal ultrasound, ventriculomegaly

## Abstract

**Background:**

The purpose of our study was to compare the incidence of chromosomal abnormalities of fetuses with isolated fetal ventriculomegaly (VM) to that of fetuses with the sole risk factor of being born to mothers of advanced age.

**Method:**

This prospective study included two groups. Group 1 included fetuses with isolated VM and were further categorized according to maternal age, fetal gender, laterality of VM (unilateral or bilateral), evolution of VM (resolved or persistent or progressive), and the gestational age at the time of diagnosis (<28w or ≥28w). Group 2 were fetuses without any fetal structural abnormality, but maternal age was at or over 35 years.

**Result:**

Eighteen fetuses (18/231, 7.8%) with chromosomal abnormalities were identified for Group 1, and 13 fetuses (13/782, 1.7%) were identified for Group 2. When cases with mothers of advanced age were excluded from Group 1, the incidence of chromosomal abnormalities of isolated VM fetuses age was 7.2%, which is still higher than that of normal structural fetuses in mothers of advanced age (*p* < 0.001).

**Conclusion:**

The risk of chromosomal abnormalities for fetuses with isolated VM is high, especially when it is severe, bilateral, the first presence occurs in mid‐gestation and is not resolved.

## 1 INTRODUCTION

Ventriculomegaly (VM) is the most commonly identified abnormal cerebral finding by prenatal ultrasound, defined as the maximum width of the atrium of the lateral ventricle being greater than or equal to 10 mm. The incidence of associated anomalies in fetuses with VM is high, about 50% according to the previous studies. Some of these malformations could be identified by ultrasound, such as open neural tube defects; however, some may be only recognizable by MRI, such as cortical malformations. Even if VM is an isolated finding, the prognosis of the fetus is still unclear, as this is also a marker for increased risk of chromosomal abnormalities and infection. Previous studies indicated that the incidence of chromosomal abnormalities for fetuses with isolated VM is between 0% and 14.2% (Bromley, Frigoletto, & Benaceraff, 1991; Graham et al., 2001). Nevertheless, there may be imperfections of the previous prenatal technology in differentiating truly isolated VM from those associated with subtle abnormalities, let alone consideration of racial differences. Therefore, with the recent improvements and standardizations of fetal screenings, we can now better determine the incidence of chromosomal abnormalities in cases with isolated VM. The purpose of our study was to explore the presence of chromosomal abnormalities in fetuses with isolated VM on a prenatal ultrasound.

Indeed, every woman is at risk for there to be a chromosomal defect in her fetus. Therefore, it is best to base counseling on an individual estimated incidence for a chromosomal abnormality. However, there are no data available for us to reference in China; therefore, existing prenatal counseling is conducted on the basis of data from North America (Hook, Cross, & Schreinemachers, 1983) and Europe (Ferguson‐Smith & Yates, 1984).

Previous studies have suggested that the risk of chromosomal abnormality increases with maternal age and, therefore, maternal age has been reported as an independent risk factor for chromosomal analysis (Abele, Luthgens, Hoopmann, & Kagan, 2011; Park, Kwon, Kim, Kim, & Shin, 2010). In the past 20 years, the proportion of older pregnancies has been increasing gradually in China. Therefore, our study was designed to compare the incidence of chromosomal abnormalities of fetuses with isolated fetal VM and that of fetuses with the sole risk factor of advanced maternal age, with the intent of using these data for future prenatal counseling in pregnancies with isolated VM.

## 2 PATIENT AND METHODS

The study was approved by the local ethics committee. Written informed consent was obtained. It is a prospective study of pregnancies seen at our institution between January 2011 and December 2016, with patients divided into two groups. Group 1 includes fetuses with isolated VM. Group 2 includes fetuses without any fetal structural abnormalities, but maternal age of the mother was at or over 35 years. Chromosomal analysis was performed in both groups. Detailed and serial ultrasound (US) examination was used to screen for fetal structural abnormalities. We performed fetal magnetic resonance imaging (MRI) and screened for fetal infection. Isolated VM cases without identified fetal infections were enrolled into Group 1.

For inclusion, the pregnancy should be of a singleton baby and all sonographic examinations and relevant examinations were made at our institution, including the sonographic examinations at 11^+0^ to 13^+6^ weeks to acquire the sonographic measurement of the nuchal translucency (NT) and crown‐rump length for gestational age (GA) determination. All cases were excluded if a diagnosis of VM was inconsistent by same‐time US and MRI. If any fetal structural abnormality was identified in the follow‐up US or MRI, cases were then excluded from the study.

US examinations and MRIs were performed by experienced sonographers and radiologists. Sonographic measurements were performed on the trans‐ventricular plane， with the calipers positioned on the internal margin of the ventricular walls, perpendicular to the long axis of the lateral ventricle. For MRI, measurements were performed at the largest part of the ventricle on the coronal plane, with good visibility of the choroid plexuses. A single observer performed the measurements of ventricular size. Each measurement was performed three times, and the mean value was determined. VM was defined as one or both ventricles measuring 10 mm or more.

Chromosome analysis of the fetuses was performed by amniocentesis or cordocentesis. A telephone follow‐up was performed for every baby that gave to birth， and the one who was suspected of other chromosomal abnormalities will be required to undergo further genetic screening. Chromosomal abnormalities were divided into numeric and structural abnormalities. Numeric abnormalities were subdivided into autosomal and sex‐chromosomal abnormalities, and structural abnormalities were subdivided into inversion, translocation, and deletion of chromosome. With regard to structural chromosomal abnormalities, normal variations in structural chromosomal abnormalities were excluded from the abnormal cases.

### 2.1 Data and statistical analysis

The cases in Group 1 were further categorized according to maternal age, fetal gender, ventricular size, laterality of VM (unilateral or bilateral), evolution of VM (resolved or persistent or progressive), and gestational age at the time of diagnosis (<28w or ≥28w). The incidence of chromosomal abnormalities relative to these categorical variables was analyzed.

Data were analyzed using SPSS version 22 (SPSS Statistics for Windows, IBM Corporation, Armonk, NY, USA). Quantitative variables are described with means and standard deviation, while qualitative variables were reported as frequency (percentage). A chi‐square (or Fisher's exact test as appropriate) test was used to compare frequencies between categorical variables and groups with a *p*‐value ≤0.05 considered as statistically significant.

## 3 RESULTS

### 3.1 Cases in Group 1

Two hundred and thirty‐one cases met inclusion criteria of Group 1, with a mean maternal age of 30 ± 4.2 years (range: 20–40 years) and the mean gestational age at diagnosis was 30.1 ± 4.4 weeks (range: 18–40 weeks). Fetal chromosome analysis was acquired by amniocentesis in 76 (32.9%) fetuses and by cordocentesis in 155 (67.1%) fetuses. No fetus underwent further genetic screening after birth. Eighteen fetuses (7.8%) with chromosomal abnormalities were identified from the original 231 fetuses. Follow‐up prenatal US scans were available in 209 (90.7%) of the 231 cases. The other 22 cases were lost to follow‐up because of termination of the pregnancy or spontaneous pregnancy loss. There was progression of VM in 85 (40.7%) fetuses, persistence in 101 (48.3%), and in utero resolution in 23 (23/209, 11%). Table 1 shows the incidence of chromosomal abnormalities relative to the different categorical variables.

**Table 1:** Incidence of chromosomal abnormalities of isolated VM in relation to different categorical variables

Categorical variables	*n*	Chromosomal abnormalities	*p* value
Maternal age			
<35 years	166	12 (7.2)	0.610
≥35 years	65	6 (9.2)	
Ventricular size			
10–15 mm	206	15 (7.3)	0.663
≥15 mm	25	3 (12)	
Laterality			
Unilateral	147	6 (4.1)	0.011
Bilateral	84	12 (14.3)	
Evolution			
Resolution	23	0 (0)	0.295
Persistence or progression	186	16 (8.6)	
Gestational age			
<28 weeks	76	11 (14.5)	0.008
≥28 weeks	155	7 (4.5)	
Fetal gender			
Male	118	9 (7.6)	0.924
Female	113	9 (8.0)	

Note

Data in parentheses are percentages.

### 3.2 Cases in Group 2

A total of 782 normal fetuses with mothers of maternal ages at or over 35 years were included in Group 2, with the mean maternal age at 39 ± 3.1 years old (range 35–48 years). Fetal chromosome analysis was acquired by amniocentesis in 703 (89.9%) fetuses and by cordocentesis in 79 (10.1%) fetuses. No fetus underwent further genetic screening after birth. There were 13 fetuses (1.7%) with chromosomal abnormalities identified out of the 782 fetuses.

### 3.3 Comparisons of incidence of chromosomal abnormalities

When cases with advanced maternal age in Group 1 were excluded, the incidence of chromosomal abnormalities of isolated VM fetuses with younger maternal age was 7.2% (expressed in Table 1), which is higher than that of normal structural fetuses with advanced maternal age (*p* < 0.001).

Table 2 shows the incidence of numeric and structural chromosomal abnormalities in both groups, and Table 3 shows the details. Trisomy 21 was the sole autosomal numeric abnormality found in our study and was also the most commonly detected chromosomal abnormality in both groups (33.3% in Group 1% and 61.5% in Group 2). Structural chromosomal abnormalities were detected at a higher rate in Group 1 than in Group 2 (*p* < 0.001).

**Table 2:** Incidences of numeric and structural chromosomal abnormalities in both groups

	Group 1			Group 2
	Maternal age <35 years	Maternal age ≥35 years	Total	
Numeric				
Autosomal	2(0.87)	4 (1.7)	6 (2.6)	8 (1.0)
Sex chromosomal	2 (0.87)	—	2 (0.87)	1 (0.13)
Total	4 (1.7)	4 (1.7)	8 (3.5)	9 (1.2)
Structural				
Inversion	2 (0.87)	—	2 (0.87)	1 (0.13)
Translocation	5 (2.2)	1 (0.4)	6 (2.6)	2 (0.26)
Deletion	1 (0.4)	1 (0.4)	2 (0.87)	1 (0.13)
Total	8 (3.5)	2 (0.87)	10 (4.3)	4 (0.51)

Note

Data in parentheses are percentages.

**Table 3:** Details of numeric and structural chromosomal abnormalities in both groups

	Group 1		Group 2
	Maternal age <35 years	Maternal age ≥35 years	
Numeric			
Autosomal	Trisomy 21(2)	Trisomy 21(4)	Trisomy 21(8)
Sex chromosomal	45, X (2)	—	Triploid (1)
Structural			
Inversion	Chromosome 9 (1) Chromosome Y (1)	—	Chromosome 9
Translocation	Reciprocal (4) Robertsonian (1)	Reciprocal (1)	Reciprocal (2)
Deletion	Chromosome 10p (1)	Chromosome 5p (1)	Chromosome 15q (1)

Note

Data in parentheses are number of cases.

## 4 DISCUSSION

The possible relationship of VM and chromosomal abnormalities has been examined by several studies. Graham addressed that the finding of mild VM is present in 0.15% of euploid fetuses and in 1.4% of all fetuses, providing a likelihood ratio of 9 for the risk of aneuploidy (Graham et al., 2001). Gezer detected a higher incidence of chromosomal abnormalities when VM was isolated (8.6%) rather than associated with any anomaly (3.8%), suggesting that karyotype analysis should be performed in all patients (Gezer et al., 2014). Consistent with the previous results, Tomlinson (Tomlinson, Treadwell, & Bottoms, 1997) also reported an increased risk of chromosomal abnormalities in isolated VM. In the existing study, we identified 7.8% of chromosomal abnormalities (including numeric and structural abnormalities) in cases with isolated VM, with trisomy 21 being the most commonly detected chromosomal abnormality (6/18, 33.3%). We did not compare its incidence of chromosomal abnormalities with that of fetuses associated with other abnormalities. We categorized our isolated VM cases according to maternal age, fetal gender, ventricular size, laterality of VM (unilateral or bilateral), evolution of VM (resolved or persistent or progressive), and the gestational age at the time of diagnosis (<28w or ≥28w). Our results suggest that the incidence of chromosomal abnormalities is higher when it is severe (≥ 15 mm), bilateral, the first presence is found mid‐gestation, and is not resolved (although some of these differences are not statistically significantly). Previous studies (Nadel & Benacerraf, 1995; Patel, Goldstein, Tung, & Filly, 1995) have noted that the width of the lateral ventricles is statistically significantly higher in males than in females; however, we were unable to find differences of chromosomal abnormalities between male and female fetuses (*p* = 0.924).

Undoubtedly, an increase in maternal age should increase the risk of chromosomal abnormalities in fetuses with isolated VM. Our results demonstrated that the incidence of chromosomal abnormalities in the subgroup with advanced maternal age trended higher (9.2%) than in the subgroup with mothers younger than 35 (7.2%); however, the differences were not statistically significantly (*p* = 0.610). Even if we excluded the cases with isolated VM and advanced maternal age, there is a significant difference of incidence of chromosomal abnormalities between fetuses with isolated VM in those with mothers of advanced maternal age, suggesting that isolated VM should be assessed as an independent risk factor (*p* < 0.001).

Several articles have explored the incidence of chromosomal abnormalities between severe and mild VM. Nicolaides (Nicolaides, Berry, Snijders, Thorpe‐Beeston, & Gosden, 1990) reported that incidence of chromosomal abnormalities is lower in cases with severe VM than in cases with borderline VM. Conversely, and consistent with our results, Graham (Graham et al., 2001) found the chromosomal abnormality incidence was higher in severe (18.2%) than mild VM (4.2%). Additionally, the incidence of aneuploidy in fetuses with mild VM (10–15 mm) has been found to be four in 148 (2.7%) by Vergani (Vergani et al., 1998) and nine in 234 (3.8%) by Pilu (Pilu et al., 1999). However, Bromley found the incidence of aneuploidy to be five (12%) in 44 fetuses with mild VM (10–12 mm) (Bromley et al., 1991). Discrepancies of results in the above studies may be explained by their different inclusion criteria.

For comparison, we also measured the incidence of chromosomal abnormalities in the group with advanced maternal age. Indeed, it is not our intent to estimate the expected risk of chromosomal abnormalities in pregnant women over the age of 35, as this is too complicated to estimate. Instead, we compared our results to this group because advanced maternal age is understood to be the proverbial independent risk factor for chromosomal analysis and these cases were available for us to get enough data. According to our results, the incidence of chromosomal abnormalities in isolated VM is higher, even, than if the advanced maternal age was excluded from this group. We believe that our result could be used for prenatal consulting to recommend chromosome analysis in a fetus with isolated VM.

The common chromosomal abnormalities associated with VM are trisomies 13, 18, and 21 (Gezer et al., 2014; Sethna, Tennant, Rankin, & C. Robson, 2011), and those with advanced maternal age are trisomy 21, trisomy 18, XXX, and XXY (Halliday, Watson, Lumley, Danks, & Sheffield, 1995; Hook, Cross, Jackson, Pergament, & Brambati, 1988). We did not observe cases of trisomy 13 and 18, potentially due to the association with trisomy 13 and 18 to multiple malformations, which were excluded in our study. We identified one case of Cri du chat syndrome, which is a rare chromosomal disorder (occurring in 1 in 20,000 to 1 in 50,000 livebirths) that results from a partial deletion of the short arm of chromosome 5 (Stefanou, Hanna, Foakes, Crocker, & Fitchett, 2002). Prenatal diagnosis of this syndrome in association with abnormal ultrasonographic features has been reported little, among them was one case with moderate bilateral VM as the only abnormal ultrasonographic finding. In our study, it was a pregnancy of 38 years old whose fetus was found with bilateral VM at 32 gestational weeks. And in follow‐up ultrasound, the ventricular size was progressive from 11 to 13 mm.

Several study limitations are acknowledged. The first one is the limited numbers in certain groups. Therefore, we hope our study can serve as a trigger for a nationwide, multicenter observational study. The second limitation is that our control group was not comprehensive, as we did not include data of any low‐risk population. However, we were unable to acquire sufficient chromosomal information for statistical analysis with this group, because they were unwilling to undergo an amniocentesis, which comes with a higher risk of spontaneous abortion of the baby. The third limitation is that our retrospective study only uses prenatal cytogenetic analysis to analyze the fetal chromosome. We should acknowledge that some chromosomal abnormalities might be missed, such as the microdeletion, although we had a telephone follow‐up for every newborn. Finally, we did not examine other potential effect, such as the effect of paternal age.

In summary, the risk of chromosomal abnormalities for fetuses with isolated VM is high, particularly when it is severe, bilateral, first known presence in mid‐gestation and not resolved. Advanced maternal age may increase its risk of chromosomal abnormalities; however, there is no significant difference.

## CONFLICT OF INTEREST

None declared.
